# Serial Killing of Tumor Cells by Human Natural Killer Cells – Enhancement by Therapeutic Antibodies

**DOI:** 10.1371/journal.pone.0000326

**Published:** 2007-03-28

**Authors:** Rauf Bhat, Carsten Watzl

**Affiliations:** Institute for Immunology, University Heidelberg, Heidelberg, Germany; Centre de Recherche Public-Santé, Luxembourg

## Abstract

**Background:**

Natural killer cells are an important component of the innate immune system. Anti-cancer therapies utilizing monoclonal antibodies also rely on the cytotoxicity of NK cells for their effectiveness. Here, we study the dynamics of NK cell cytotoxicity.

**Methodology/Principal Findings:**

We observe that IL-2 activated human NK cells can serially hit multiple targets. Using functional assays, we demonstrate that on an average, a single IL-2 activated NK cell can kill four target cells. Data using live video microscopy suggest that an individual NK cell can make serial contacts with multiple targets and majority of contacts lead to lysis of target cells. Serial killing is associated with a loss of Perforin and Granzyme B content. A large majority of NK cells survive serial killing, and IL-2 can replenish their granular stock and restore the diminished cytotoxicity of ‘exhausted’ NK cells. IL-2 and IL-15 are equally effective in enhancing the killing frequency of resting NK cells. Significantly, Rituximab, a therapeutic monoclonal antibody increases the killing frequency of both resting and IL-2 activated NK cells.

**Conclusion/Significance:**

Our data suggest that NK cell-based therapies for overcoming tumors rely on their serial killing ability. Therefore, strategies augmenting the killing ability of NK cells can boost the immune system and enhance the effectiveness of monoclonal antibody-based therapies.

## Introduction

Natural killer cells are key players in eliminating and preventing tumor growth [1]. They execute their cytotoxicity in a sequential manner, which involves (i) formation of conjugate between NK and tumor cell (ii) delivery of lethal hit (iii) disassociation of tumor and NK cell. A disassociated NK cell can restart and bind to new targets and finally eliminate them, commonly referred to as recycling capacity or killing frequency [2]. In case of cytotoxic T lymphocytes, it has been demonstrated that at a single cell level a mouse effector lymphocyte can kill one target and has the potential to recycle and kill up to six targets in a sequential manner [3]. Computational models have attempted to explain kinetic parameters of recycling capacity of effector cells [2], [4], [5]. Nevertheless, only little information exists about killing potential or frequency of NK cells. How often, and how many targets a single human NK cell can kill is still unknown. Knowledge regarding the frequency of NK cell killing can prove helpful for devising novel strategies to boost NK cell efficiency.

NK cells undergo functional anergy or exhaustion after carrying out their killing action. Previous studies have demonstrated that after an exposure to target cells, NK cells undergo inactivation, loose their cytotoxic function and become apoptotic [6], [7]. Cytotoxicity of NK cells is executed mainly through the granule exocytosis pathway where Perforin and Granzyme B content of granules is released into the immunological synapse after conjugate formation with targets [8]. This results in a depletion of these components and in a loss of cytotoxicity. Whether the cytotoxic function and granular content of NK cells, which have already participated in serial killing and have undergone inactivation can be reversed is still unknown.

Strategies to augment NK cell activity for tumor immunotherapy are actively pursued at present [9], [10]. IL-2, IL-15 and IFN-α have been implicated in NK cell activity, growth, development and differentiation [11]–[13]. IL-15 has been shown to be a major factor in development, increasing cytotoxicity and proliferation of NK cells. Similarly, IL-2 has been used effectively in cancer immunotherapy in clinical trials. IFN-α has been shown to promote NK cell mediated cytotoxicity and defense against viral infections. Stimuli that can enhance killing potential and frequency of NK cells still need to be identified.

Studies exploring therapeutic monoclonal antibodies in potentiating host immune responses against malignancies are being pursued vigorously and have been successful [14]. Rituximab, one widely studied therapeutic monoclonal antibody, is a chimeric IgG1 monoclonal antibody that specifically targets CD20 surface antigen expressed on normal and neoplastic B-lymphoid cells [15]. Rituximab has been shown to induce apoptosis, complement mediated lysis and antibody-dependent cellular cytotoxicity in vitro. The effectiveness of rituximab relies on the cytotoxic function of NK cells [9], [10], [16], [17]. Whether Rituximab enhances the frequency of killing by NK cells is still unexplored.

In the present study we investigate the number of targets a single IL-2 activated NK cell can eliminate. Our findings demonstrate that similar to CTLs, NK cells can make multiple contacts and kill serially in a time-dependent manner. A large majority of NK cells survive after such serial killing and IL-2 has the potential to restore the cytotoxicity and granular content of exhausted NK cells. Rituximab treatment enhances the killing frequency of fresh and IL-2 activated NK cells. Strategies resulting in increased serial killing of NK cells can therefore enhance the anti-tumor activities of these strategically important immune cells.

## Results

### NK cells can kill multiple targets

**Figure 1:**
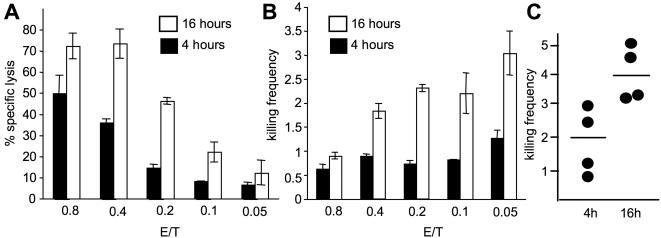
IL-2 activated human NK cells can kill multiple targets. IL-2 activated primary human NK cells were co-incubated with ^51^Cr labeled 221 target cells at an E∶T ratio of 0.8∶1 to 0.05∶1 for 4 and 16 hours. (A) 51 Cr release assay was used to calculate % specific lysis for 4 and 16 hours duration. (B) % specific lysis was used to calculate killing frequency by dividing the number of target cells killed by number of effector cells used for each E∶T ratio. (C) Killing frequency of IL-2 activated NK cells using four independent donors for 4 and 16 hours duration.

**Figure 2:**
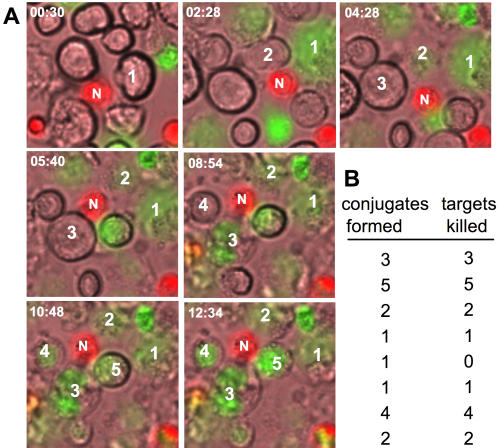
Serial killing by an NK cell. (A) A single NK cell makes multiple contacts with tumor cells resulting in lysis of tumor cells. Dye-labeled IL-2 activated NK cells (N, red) were incubated with unlabelled 221 tumor cells at an E∶T ratio of 0.2∶1 for 16 hours at 37°C and 5% CO2 and images were recorded by time lapse microscopy. Dead cells were detected using Sytox Green and numbered in the order of lysis. Time (h:mm) is indicated in the upper left corner of each image. (B) Majority of conjugate formations result in lysis. Conjugate formations between NK and 221 cells were randomly selected and lysed target cells counted. To rule out the possibility of interference of dyes in killing process, a control ^51^Cr release assay was performed with labeled cells. No difference was noted between the killing ability of labeled and unlabeled cells (data not shown).

To explore the maximal killing potential of NK cells, we first identified a tumor target most susceptible to NK cell lysis. We therefore tested several target cell lines against IL-2 activated NK cells and found that MHC-I negative 722.221 cells showed better lysis that K562 targets and were most susceptible to NK cell killing (data not shown). As effector cells we chose to use primary human NK cells from healthy donors, which had been expanded in IL-2 for two weeks, as these cells showed maximal cytotoxic activity. To determine the number of targets killed per individual NK cell, we used a ^51^Cr release assay with an excess of target cells. This way each NK cell has access to a large number of targets, making it possible to estimate the maximal killing activity. We performed standard 4 h and also 16 h ^51^Cr release assays to determine the full potential of NK cell activity. Killing activity of NK cells was assessed in terms of % specific lysis and the amount of target cells lysed per NK cell (killing frequency) was determined by dividing the number of killed targets by the number of effector cells (Fig. 1). Killing frequency was inversely proportional to % specific lysis and increased from higher to lower effector to target ratios. This is probably be due to a greater possibility for an individual NK cell to form contacts with target cells at lower effector to target ratios. Analysis of the killing frequency of NK cells from multiple donors demonstrates that on an average two or four targets are killed by an NK cell after 4 or 16 hours, respectively (Fig. 1C). This suggests that IL-2 activated NK cells are capable of making multiple contacts and can kill multiple targets. To visualize the process of serial killing, we employed time lapse video microscopy (Fig. 2). Dye labeled NK cells were incubated with unlabeled 221 target cells and observed under 16 hour time lapse microscopy. Sytox dye was used to visualize dead cells. These data confirm that a single NK cell can make serial contacts with multiple targets and finally kill them. Most of the contact formations observed resulted in killing (Fig. 2B). However, all NK cells were not equally active in forming contacts and killing targets but few ‘hyperactive’ NK cells were also observed, lysing up to 6 individual targets (Fig. 2 and data not shown). Interestingly, during the process of killing only very few dead NK cells were detected.

### NK cells undergo a reduction of Perforin and Granzyme B but survive serial killing

**Figure 3:**
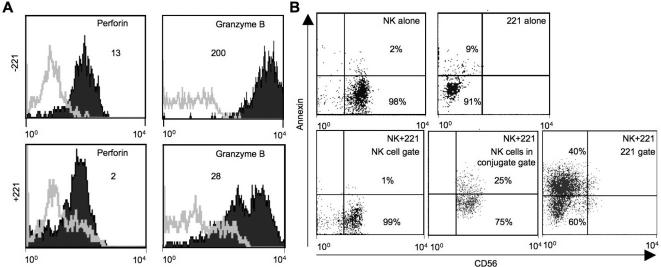
Serial killing of tumor cells by NK cells is mediated by Perforin and Granzyme B. (A) IL-2 activated NK cells, alone (−221) or together with 221 target cells (+221) were incubated for a period of 16 hours at an E∶T ratio of 0.2∶1 and then stained intracellularly for Perforin and Granzyme B content. NK cells were identified by gating on CD56 positive cells. Fold expression shown here represents the ratio of MFI of Perforin/Granzyme B and respective isotypes. Figure is representative of multiple donors. (B) IL-2 activated NK cells were incubated alone or in combination with 221 targets at an E∶T ratio of 0.2∶1. After 16 hours, cells were harvested and stained for CD56 expression (NK cells) and annexinV (apoptotic cells). NK and 221 cells alone were used as controls. Forward and sideward scatter gating was used to gate on free NK cells, free 221 cells or conjugates. Figure is representative of multiple donors.

Cytotoxicity of NK cells is mostly mediated by the release of Perforin and Granzyme B [8]. To determine whether serial killing is granule-mediated, we analyzed Perforin and Granzyme B expression in NK cells after co-incubation with 221 cells using intracellular flow cytometry. Data from multiple donors showed that there was significant reduction in Perforin and Granzyme B content after target cell contact (Fig. 3A). The decrease was more prominent in case of Granzyme B than Perforin in all cases observed. Interestingly, we never observed a complete loss of these molecules even after prolonged target cell contact (data not shown). Overall, these data suggests that serial killing by NK cells is mediated by Perforin and Granzyme B.

To investigate whether NK cells survive or undergo apoptosis after killing their targets, NK cells were incubated alone and with 221 targets for 16 hours. After incubation, cells were harvested and stained for CD56 expression and with AnnexinV to identify apoptotic cells (Fig. 3B). Gating on free NK cells showed only a negligible fraction of apoptotic cells after target cell contact, while a significant number of 221 cells were clearly apoptotic. However, when gating on the conjugates between NK and 221 cells, we found around 25% of NK cells to be AnnexinV positive. Overall we found 15% of total NK cells (free and in conjugates) to be apoptotic after 16 h target cell contact. The large number of apoptotic NK cells in the conjugate gate could be an artifact of the conjugates where the NK cell contributes for the CD56 staining, but only the attached 221 cell is AnnexinV positive. We therefore treated the conjugates with EDTA for 15 min. at 37°C to dissociate them. EDTA treatment resulted in approximately 50% of conjugates falling apart and also reduced the number of AnnexinV positive NK cells (data not shown). This demonstrates that at least some NK cells only appeared to be apoptotic because of an attached AnnexinV positive 221 cell. However, a fraction of NK cells still remained AnnexinV positive. Taken together, these results suggest that majority of NK cells survive the killing process and only a small number of NK cells undergo apoptosis and these cells lie in the conjugate region.

### IL-2 restores cytotoxicity and granular stock in NK cells

**Figure 4:**
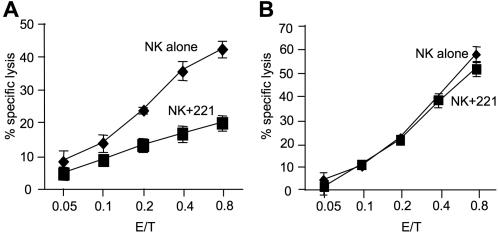
IL-2 restores the killing ability of inactivated NK cells. IL-2 activated NK cells were incubated with or without 221 targets for 16 hours at an E∶T ratio of 0.2∶1. Thereafter, NK cells were re-isolated by CD56 positive selection using MACS columns and cultured in the presence of IL-2 (100 U/ml). Killing assays against 221 target cells were performed on day 1 (A) and 2 (B) after isolation. Data from three independent donors show that NK cells regain their lost cytotoxic ability on day 2 in response to IL-2 treatment.

As NK cells experience a reduction in the amount of Perforin and Granzyme B after serially killing of tumor cells but survive the process, we wanted to investigate whether the cytolytic ability of these NK cells can be restored. We therefore incubated NK cells alone or in combination with 221 cells for 16 hours. After that the cells were harvested and CD56 positive NK cells were re-isolated using MACS positive selection. The isolated NK cells were then cultured in presence of IL-2 and killing activity was examined. One day after isolation the NK cells recovered after target cell incubation demonstrated a significant lower cytotoxicity against 221 target cells as compared to cells isolated from the control fraction (Fig. 4A). However, the killing ability of these ‘exhausted’ NK cells was augmented in response to IL-2 treatment on day 2 and was similar to that of control NK cells (Fig. 4B). We also determined Perforin and Granzyme B staining of these NK cells. Perforin and Granzyme B content showed an increase after isolation and culture with IL-2, correlating with the observed increase in killing activity (data not shown).

### IL-2 and IL-15 are equally effective in increasing cytotoxicity of NK cells

**Figure 5:**
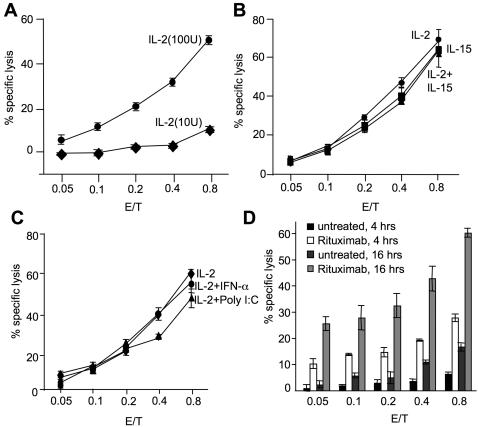
Increasing the killing frequency of fresh NK cells by cytokines and Rituximab. (A) Freshly isolated NK cells were cultured with IL-2 (10 or 100 U/ml) alone (A) or with IL-2 (100 U/ml) and IL-15 (10ng/ml), alone or in combination (B) IL-2 (100 U/ml) alone or in combination with IFN-α (1000U/ml) or poly I∶C (50 µg/ml) (C) for one week. Thereafter, stimulated NK cells were tested against 221 target cells using 4 hours Cr-^51^ release assays. Combining IFN-α or poly I∶C with only 10 U/ml IL-2 also showed no enhancement of cytotoxicity (data not shown). (D) 221 target cells were treated with Rituximab (10 µg/ml) and killing frequency of freshly isolated NK cells was assessed by 4 and 16 h ^51^Cr release assay and compared to untreated targets. Data are representative of three independent experiments using NK cells from different donors.

In the experiments described above we used primary human NK cells, which had been expanded in the presence of IL-2 for 2 weeks, as such cells showed a high cytotoxic activity. To test whether other factors can modulate NK cell killing activity and subsequently killing frequency, we stimulated freshly isolated human NK cells with IL-2, IL-15, IFN-α or polyI∶C for 7 days and assessed their killing activity. Cells stimulated with low concentrations of IL-2 (10 IU/ml) were found to be poorly lytic compared to cells cultured in the presence of 100 IU/ml of IL-2 (Fig. 5A). This suggests that while low doses of IL-2 are sufficient for the survival of NK cells, only higher doses can efficiently stimulate their cytotoxicity. Next we stimulated NK cells with IL-2 or IL-15. Both cytokines were found to be equally effective in increasing the cytolytic ability of NK cells (Fig. 5B). However, no synergistic effect was observed when IL-15 and IL-2 were used in combination. IFN-α, polyI∶C or CpG oligo nucleotides either alone or in combination with IL-2 failed to induce any significant difference in NK cell cytotoxicity when compared to IL-2 treatment alone (Fig. 5C and data not shown).

### Rituximab increases killing frequency of NK cells

Monoclonal antibodies used for anti-cancer therapies can effectively stimulate NK cell activity through the Fc receptor CD16 [16], [17]. To investigate whether a therapeutic antibody can increase the killing frequency of freshly isolated NK cells, we incubated 221 cells with Rituximab and used them as targets for NK cells in a 4 and 16 h ^51^Cr release assay (Fig. 5D). While freshly isolated NK cells were only poorly cytotoxic against control treated 221 cells, their cytotoxic activity could be greatly enhanced by using Rituximab-coated 221 cells as targets. This is consistent with a previous report demonstrating that CD16 is sufficient to trigger the cytotoxic activity of resting NK cells [18]. The use of Rituximab-coated 221 cells resulted in the lysis of 2–3 targets per NK cell in a 16 h assay. This demonstrates that even freshly isolated NK cells have cytotoxic potential and are capable of serial killing.

**Figure 6:**
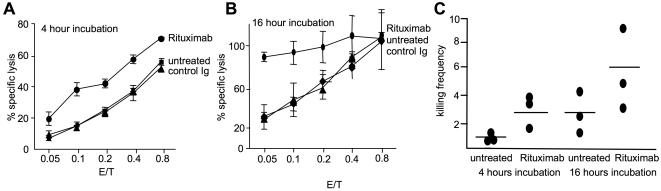
Rituximab increases killing frequency of IL-2 activated NK cells. 221 target cells were treated with Rituximab (10 µg/ml) and killing frequency of NK cells was assessed by 4 h (A) or 16 h (B) ^51^Cr release assay and compared to untreated or isotype treated targets. (C) Data from three independent donors show that rituximab increases the killing frequency of NK cells two fold as compared to untreated target cells.

Furthermore, we wanted to test if Rituximab could also enhance the killing frequency of IL-2 activated NK cells, which were already highly cytotoxic (Fig. 1). Rituximab treatment resulted in a significant increase of NK cell cytolytic ability and consequently also killing frequency, which was already obvious after 4 hours, but most prominent after 16 hours of incubation with IL-2 activated NK cells (Fig. 6). Data from three independent donors showed an increase of two fold in killing frequency as compared to untreated target cells, reaching a maximum of 6 target cells killed per NK cell after 16 hours (Fig. 6C). Control antibodies showed no effect. We also analyzed the effect of Rituximab treatment on the decrease of Perforin and Granzyme B content using intracellular staining. Data from three donors showed further reduction in Perforin and Granzyme B expression on coincubation with rituximab treated targets as compared to untreated targets (data not shown).

## Discussion

Natural killer cells are known to participate actively in combating tumors. However, details about dynamics of NK cell cytotoxicity are still lacking. How much potential a singe NK cell has to kill and the number of hits it can deliver when encountered by multiple targets is still unknown. Previous reports have attempted to unravel the killing frequency of mouse CTLs under controlled conditions and observed that a single CTL can eliminate up to six targets [3]. Later various computational models, utilizing various variables, were employed to deduce kinetics of binding of NK cells to multiple targets and their recycling capacity, but no consensus exists [2], [4], [5]. Moreover, these reports do not give any idea about the killing frequency of a single NK cell. Our study attempts to reveal the maximal potential of IL-2 activated human NK cells in eliminating tumor cells.

From the ^51^Cr release data we conclude, that a single IL-2 activated NK cell can kill four 221 target cells in a 16 hour assay. This calculation assumes, that the release of ^51^Cr is an accurate measure about the number of target cells lysed. However, we cannot exclude, that there is also unspecific or bystander lysis in our assay, which would lead to an over estimation of the number of target cells killed. We therefore employed time lapse microscopy to directly demonstrate the serial killing of NK cells. Although we did observe individual NK cells serially killing 4 or even more targets, many NK cells only killed once or twice. It is not possible to directly calculate the average killing capacity of NK cells from this assay because of the experimental setup of the microscopy. We could only use a limited concentration of cells in order to clearly observe individual NK-target cell interactions. Therefore, not every NK cell has even the chance to meet four or more targets in this assay and we cannot expect to see the full killing potential of all NK cells. However, most of the conjugate formations with the target cells resulted in lysis. This is in contrast to a recent report, demonstrating that almost 50% of the conjugates between IL-2 activated mouse NK cells and YAC-1 targets are non-productive and dissociate without killing [19]. The reason for this discrepancy might be the choice of target or the species origin of the NK cells.

The ^51^Cr release data can only give an average killing frequency as the activity of many NK cells is assayed. In our time lapse microscopy we observed some hyperactive NK cells, which were more fervent in their killing spree, while other NK cells were much more stationary, killing only one target. One possible explanation for this observation is that there is heterogeneity among NK cells, with some cells being much more active than others. This is also reflected by the analysis of Perforin and Granzyme B content, where we observed that some NK cells suffered a greater loss of these effector molecules after target cell contact (Fig. 3A), possibly due to higher activity and more lysis. It will be interesting to analyze the nature of such hyperactive NK cells. This could lead the way for selecting NK cells with especially high activity for NK cell based therapies or enable us in the future to better stimulate NK cell activity.

We observed that a single NK cell could make contacts to two or more targets at the same time. However, the serial killing was found to operate strictly in a sequential manner as we never observed any simultaneous killing of two or more targets by a single NK cell. Some NK cells also stayed attached to target cells after lysis was complete. This was also observed in our FACS analysis where we detected NK cells in conjugate with AnnexinV positive target cells. It is interesting to speculate that the multiple target cell contacts before and after lysis could enhance NK cell activation by providing additional activating receptor engagement and NK cell stimulation. Such uncoupling of lytic and stimulatory contacts has recently been shown for cytotoxic T lymphocytes [20].

NK cells underwent a significant reduction in their Perforin and Granzyme B content after target cell incubation. The loss of Perforin during a single degranulation event of resting NK cells was reported to be rather small [21]. Even four of such degranulation events might not explain the significant reduction in Perforin and Granzyme B we observed in our experiments. One explanation for this might be that IL-2 activated NK cells are more active and release much more cytotoxic granules during a single degranulation event. We never observed a complete loss of Perforin and Granzyme B in exhausted NK cells. This demonstrates that also other mechanisms besides the loss of Perforin and Granzyme B are the reason for NK cell exhaustion. Such mechanisms could be the down-modulation of activating receptors [22] or the inactivation of signaling pathways due to constant stimulation. The majority of such exhausted NK cells survived the target cell contact and could even be re-activated by IL-2 stimulation after separation from target cells. This suggests that IL-2 is necessary not only for maintenance and survival of NK cells but also important for the regeneration of inactivated NK cells. The separation of NK cells from target cells may be crucial as a previous report suggested that the cytotoxic activity of inactivated NK cells could be enhanced by overnight IL-2 treatment when NK cells were dissociated from conjugates while this was not possible with associated conjugates [7]. This is in line with our finding that most apoptotic NK cells were found in conjugate with 221 cells. Also an earlier study demonstrated that inactivated and dead NK cells are restricted to conjugates between NK and target cells [6]. The removal of apoptotic cells by phagocytic cells *in vivo* may therefore play an important role in keeping NK cells active.

Our and many previous data show that IL-2 is a very potent stimulator of NK cell cytotoxicity. While IL-15 showed a similar potency, we did not observe any synergistic effect when combining IL-2 and IL-15. Also using IL-2 in combination with polyI∶C or CpG oligonucleotides to stimulate TLRs did not result in an increased activity of NK cells. One explanation for this could be that IL-2 or IL-15 induce already a maximal stimulation of NK cells. However, fresh and IL-2 stimulated NK cells could be further stimulated by engagement of CD16 through rituximab coated target cells. It was surprising to see such an enhancement, as the 221 cells represent already the most sensitive target cell for IL-2 activated NK cells in our hands. The rituximab-mediated enhancement of lysis was already very obvious in a 4 h assay. As we do not expect to see any significant exhaustion of NK cells during this short time frame, we speculate that rituximab either enhances the speed by which an individual NK cell can lyse, or stimulate previously inactive NK cells to kill. Also after 16 hours we observed a clear enhancement of lysis by rituximab, almost doubling the killing frequency of IL-2 activated NK cells. This effect could again be mediated by stimulating previously inactive NK cells. However, it is likely that rituximab increases the potential of NK cells to hit multiple targets. In line with this hypothesis we observed that rituximab treatment enhanced degranulation of NK cells as evident by further reduction in Perforin and Granzyme B content of NK cells. These data suggest that therapeutic monoclonal antibodies against tumors may rely on the serial killing of NK cells and strategies focusing on enhancing the killing potential of NK cells are likely improve their effectiveness.

## Materials and Methods

### Cell culture, cell lines and cytokines

Human polyclonal NK cells were isolated by negative depletion from peripheral blood lymphocytes (PBLs) using an NK-cell isolation kit (Dynal, Karlsruhe, Germany). NK cells were between 90% and 99% CD3^−^ and CD56^+^. NK cells were expanded in the presence of IL-2 and feeder cells as described (18,19). 721.221 cells were grown in IMDM, 10% fetal calf serum, penicillin/streptomycin (Invitrogen, Karlsruhe, Germany). Fresh NK cells were stimulated without feeder cells in the presence of IL-2 (100 IU/ml) (NIH cytokine repository), IL-15 (10 ng/ml) (R&D systems, Wiesbaden, Germany) and IFN-α (1000 IU/ml) or poly I∶C (50 µg/ml) (Sigma-Aldrich, Taufkirchen, Germany) for 1 week.

### FACS analysis and antibodies

For surface staining, the cells were incubated with anti-CD56 PE or FITC and CD19 FITC Abs (BD Biosciences, Heidelberg, Germany) in 50 µl of FACS buffer (PBS, 2% FCS) for 20 min on ice. All washing steps were performed with cold FACS buffer. For intracellular staining, cells were first stained with cell surface mAb as described, washed, and then fixed with 4% paraformaldehyde for 15 min and permeabilized in PBS, 2% FCS, 0.5% saponin and incubated with Perforin-FITC (BD Pharmingen, Heidelberg, Germany) and Granzyme B PerCP (Caltag laboratories) for 30 min at 4°C. Cells were then washed and immediately analyzed on a FACSCalibur. For analysis of apoptosis, cells were harvested, surface stained, washed and centrifuged (1500 rpm, 5 min). The cell pellets were suspended in 1×binding buffer (10 mM HEPES, pH 7.4, 140 mM NaCl, 2.5 mM CaCl_2_). The cells were stained using 5 µl each of annexin V-PE (BD Pharmingen, Heidelberg, Germany), gently vortexed and incubated for 15 min at room temperature. 400 µl of binding buffer was added before FACS analysis.

### Cytotoxicity assay

Target cells were grown to mid-log phase, and 5×10^5^ cells were labeled in 100 µl of assay medium (IMDM with 10% FCS and penicillin/streptomycin) with 100 µ Ci ^51^Cr for 1 hr at 37°C. Cells were washed twice and resuspended in assay medium. A total of 5000 target cells/well was used in the assay. Effector cells were resuspended in assay medium, distributed on a V-bottom 96-well plate, and mixed with labeled target cells at different E∶T ratios. Maximum release was determined by incubating target cells in 1% Triton X-100. For spontaneous release, targets were incubated without effectors in assay medium alone. In case of rituximab treatment, target cells were incubated with Rituximab (Roche, Mannheim, Germany) or a control antibody (MOPC-21 (Sigma, Taufkirchen, Germany)) at a final concentration of 10 µg/ml. All samples were done in triplicates, and IL-2 was used at 100 IU/ml final concentration in the whole assay. After a 1 min centrifugation at 1000 rpm, plates were incubated for 4 and 16 h at 37°C. Supernatant was harvested and ^51^Cr release was measured in a gamma counter. Percentage of specific release was calculated as (experimental release-spontaneous release)/(maximum release-spontaneous release) ×100.

### Time-lapse video microscopy

NK (1×10^6^) cells were harvested, washed twice and resuspended in 100 µl of diluent C (Fluorescent Red Linker Kit, Sigma, Taufkirchen, Germany). Red dye (1∶50), provided with the kit, was added and cells incubated at RT for 5 min. 100 µl of heat-inactivated FCS was added and incubated for 1 min to stop the reaction. Thereafter, cells were washed with RPMI+10%FCS thrice to remove unlabelled dye and then incubated for 2 hours at 37°C. After that cells were counted and co-incubated with unlabelled 221 cells. Sytox Green nucleic acid stain (Invitrogen, Karlsruhe, Germany) at a final concentration of 100 nm/ml was used to detect dead cells. Cells were co-incubated in micro-chambers (Lab-Tek Chambers cover-glasses, Nalgene Nunc, Wiesbaden, Germany) and observed under Zeiss LSM-510 Time Lapse microscope at 37°C and 5% CO_2_. Image sequences of time-lapse recording were processed with METAMORP software.
